# Adoption of the 2A Ribosomal Skip Principle to Tobacco Mosaic Virus for Peptide Display

**DOI:** 10.3389/fpls.2017.01125

**Published:** 2017-06-28

**Authors:** Juliane Röder, Rainer Fischer, Ulrich Commandeur

**Affiliations:** Institute for Molecular Biotechnology, RWTH Aachen UniversityAachen, Germany

**Keywords:** tobacco mosaic virus, ribosomal skip, fluorescent protein, iLOV, peptide display, nanoparticles

## Abstract

Plant viruses are suitable as building blocks for nanomaterials and nanoparticles because they are easy to modify and can be expressed and purified using plants or heterologous expression systems. Plant virus nanoparticles have been utilized for epitope presentation in vaccines, for drug delivery, as nanospheres and nanowires, and for biomedical imaging applications. Fluorescent protein fusions have been instrumental for the tagging of plant virus particles. The monomeric non-oxygen-dependent fluorescent protein iLOV can be used as an alternative to green fluorescent protein. In this study, the iLOV sequence was genetically fused either directly or via a glycine-serine linker to the C-terminus of the Tobacco mosaic virus (TMV) coat protein (CP) and also carried an N-terminal Foot-and-mouth disease virus (FMDV) 2A sequence. *Nicotiana benthamiana* plants were inoculated with recombinant viral vectors and a systemic infection was achieved. The presence of iLOV fusion proteins and hybrid particles was confirmed by western blot analysis and transmission electron microscopy. Our data suggest that TMV-based vectors are suitable for the production of proteins at least as large as iLOV when combined with the FMDV 2A sequence. This approach allowed the simultaneous production of foreign proteins fused to the CP as well as free CP subunits.

## Introduction

Plant virus capsids are highly suitable for nanotechnological applications because they can be modified by genetic engineering or chemical conjugation to exposed amino acid residues. The capsids comprise multiple copies of one or more identical CP subunits, thus providing many different possibilities for the selective attachment and presentation of numerous organic and inorganic molecules, including metals, semi-conductors, carbohydrates, peptides and larger proteins such as antibodies. Various molecules have already been displayed on the surface of CPMV and TMV capsids (Meunier et al., 2004; Blum et al., 2005; Chatterji et al., 2005; Steinmetz et al., 2006a,b; Niu et al., 2007; Liu et al., 2012; Brown et al., 2013). Therefore, plant viruses are suitable as building blocks for applications in areas as diverse as electronics and vaccination. Fluorescence-tagged viral nanoparticles can also be used as biomedical imaging devices (Lewis et al., 2006; Manchester and Singh, 2006; Brunel et al., 2010; Wen et al., 2012; Lee et al., 2013; Shukla et al., 2014). In this context, the filamentous PVX has proven superior to spherical CPMV particles because PVX achieves enhanced tumor homing and retention and can carry a larger payload (Shukla et al., 2013).

TMV, the type member of the genus *Tobamoviruses* (family *Virgaviridae*), is one of the best-characterized plant viruses and is a popular choice for nanotechnological applications (Dujardin et al., 2003; McCormick et al., 2006; Bruckman et al., 2008; Royston et al., 2008; Azucena et al., 2012; Luckanagul et al., 2012; Bruckman and Steinmetz, 2014). The 6.4-kb RNA genome of TMV has a plus-sense polarity, a 5′-methylguanosine cap and a tRNA-like structure at the 3′ end (Zimmern, 1977; van Belkum et al., 1985). The RNA is encapsidated by 2130 identical helically assembled CP subunits to form a rigid rod-shaped particle (Klug, 1999). Small peptides can be displayed on the surface of TMV by inserting the coding sequence at either the 5′ or 3′ end of the *cp* gene (Bloomer et al., 1978). There are further potential insertion points between amino acid positions 63 and 66 (Turpen et al., 1995) and close to the C-terminus between positions 154 and 155 (Bendahmane et al., 1999). Protein fusions can also be generated using a leaky stop codon (Skuzeski et al., 1991). For PVX, an alternative methodology to display a target protein is the insertion of the FMDV 2A sequence between the target gene and the *cp*, hence a ribosomal skip occurs during translation, leading to the expression of the CP fusion protein, free target protein as well as wild-type CP (Cruz et al., 1996; Donnelly et al., 2001a,b). The FMDV 2A sequence has previously been used in PVX vectors to avoid unfavorable limitations of CP fusions (Cruz et al., 1996; Lee et al., 2014; Shukla et al., 2014; Uhde-Holzem et al., 2015), but to our knowledge it has not yet been used in a TMV CP fusion protein.

Fluorescent proteins such as GFP are widely used as reporters in prokaryotic and eukaryotic cells, but suffer from disadvantages such as their complex structure, large size, and their dependence on pH and oxygen (Shaner et al., 2007; Gawthorne et al., 2012). A suitable alternative is the improved LOV2 (light, oxygen or voltage sensing) domain of *Arabidopsis thaliana* phototropin 2, designated iLOV (Chapman et al., 2008). A flavin mononucleotide serves as the chromophore within the LOV domain. The 13-kDa monomeric iLOV protein is 113 amino acids in length (Christie et al., 2012). Chapman et al. (2008) have already shown that the reversible photobleaching properties of iLOV make it particularly suitable as a reporter for TMV infection and movement.

In order to display iLOV on the surface of TMV, we genetically fused the iLOV sequence to the *cp* gene and visualized the systemic spread of recombinant viruses by *in planta* fluorescence monitoring. We found that viruses carrying a direct fusion of iLOV and CP initially remained predominantly in the stem, but those with an additional FMDV 2A sequence achieved a rapid systemic infection. As previously observed for analogous PVX vectors, the iLOV-2A-CP_TMV_ construct resulted in the expression of an iLOV-CP_TMV_ fusion protein, free CP_TMV_ and free iLOV. TMV particles presenting iLOV were identified by immunogold electron microscopy. Therefore, our data confirm that TMV vectors containing the FMDV 2A sequence are suitable for the display of proteins at least as large as the 13-kDa monomeric iLOV protein, which represents a protein that usually has limitations for fusions to the TMV CP due to the amino acid sequence.

## Materials and Methods

### Vector Construction

The pSC1001a vector containing the iLOV sequence was a kind gift from S. Chapman (The James Hutton Institute, Dundee, Scotland). The PVX-based vector pPVX-iLOV-2A-CP_PV X_ was constructed by amplifying the iLOV sequence using specific primers NheI-iLOV and iLOV-BspEI, which also introduced the named restriction sites (**Table 1**). The PCR products were inserted into the pCR2.1-TOPO cloning vector and introduced into competent *Escherichia coli* TOP10 cells according to the manufacturer’s recommendations (Thermo Fisher Scientific, Karlsruhe, Germany). A control PCR using the M13 forward and reverse primers was carried out to confirm the presence of the target gene. The iLOV sequence was digested with NheI and BspEI and transferred to vector pTCXIIc (Shukla et al., 2014) which was linearized with the same enzymes to remove the mCherry sequence, leaving the FMDV 2A sequence as a ligation target. The integrity of the final plasmid was confirmed by PCR using primers TGB-fw and CX1.

**Table 1:** Oligonucleotides used for PCR.

Primer name	Nucleotide sequence (5′–3′)
2A-rev	CCCGGGGTTGGACTCGACGTCT
CPTMV-G4S	GCTACCGCCTCCACCACTCCCTCCACCGCCGCTACCTCCACCTCCAGTAGCCGGAGTTGTG
CPTMV-iLOV	CTGGACCACAACTCCGGCTACTGCAAGCATAGAGAAGAATTTCGTC
CPTMV-rev	AGTAGCCGGAGTTGTGGTCCAG
CPTMV-Stop-NotI	ACCGCGGCCGCTTAAGTAGCCGGAG
CX1	TTGAAGAAGTCGAATGCAGC
G4S-iLOV	GGAGGTGGAGGTAGCGGCGGTGGAGGGAGTGGTGGAGGCGGTAGCGCAAGCATAGAGAAGAATTTC
iLOV-BspEI	TTTTCCGGATACATGATCACTTCCATCGA
iLOV-Stop-NotI	TTTGCGGCCGCTTATACATGATCACTTCCATCGAGC
M13 reverse	ACACAGGAAACAGCTATGAC
M13 forward	GTTGTAAAACGACGGCCAGT
N-2A-CPTMV	GCTTGCGGGAGACGTCGAGTCCAACCCCGGGCCTTATACAATCAACTCTCCG
NheI-iLOV	AAAGCTAGCATGGCAAGCATAGAGAAGAA
PacI-CPTMV	AAATTAATTAAATGCCTTATACAATCAACTCTC
PacI-iLOV	AAATTAATTAAATGGCAAGCATAGAGAAGAATTTCGTCATCACTG
TGB-fw	AAGGGCCATTGCCGATCTCAAGC
TMV5482f	TTGATGAGTTCATGGAAG
TMV6269r	TTCGATTTAAGTGGAGGG

The TMV-based vectors containing the iLOV sequence were constructed by splice overlap extension (SOE) PCR. The TMV *cp* sequence was amplified from vector pET22b-TMVCP-His (unpublished data) using primers PacI-CPTMV and CPTMV-rev or PacI-CPTMV and CPTMV-G4S, whereas the iLOV sequence was amplified from vector pPVX-iLOV-2A-CP_PV X_ using primers CPTMV-iLOV and iLOV-Stop-NotI or G4S-iLOV and iLOV-Stop-NotI. The primer combinations were chosen according to whether or not the linker sequence was required. The resulting CP and iLOV fragments were combined and re-amplified with flanking primers in two subsequent PCRs. The CP_TMV_-iLOV and CP_TMV_-G_4_S-iLOV fusion sequences were inserted into vector pCR2.1-TOPO for the transformation of competent *E. coli* TOP10 cells as described above. The integrity of the vectors was confirmed by PCR using the M13 forward and reverse primers. The pCR2.1 CP_TMV_-iLOV and pCR2.1 CP_TMV_-G_4_S-iLOV vectors were digested with PacI and NotI and the inserts were transferred to vector pTRBOc (Lindbo, 2007) which had been linearized with the same enzymes, thus fusing the iLOV sequence to the C-terminus of the TMV CP either directly or via the (G_4_S)_3_ linker. The integrity of the final vectors pTMV-CP_TMV_-iLOV and pTMV-CP_TMV_-G_4_S-iLOV was confirmed by PCR using primers TMV5482f and TMV6269r.

Vector pTMV-iLOV-2A-CP_TMV_ was constructed by amplifying the iLOV sequence from pPVX-iLOV-2A-CP_PV X_ using primers PacI-iLOV and 2A-rev, whereas the TMV CP sequence was amplified using primers N-2A-CPTMV and CPTMV-Stop-NotI. The fragments were joined by SOE-PCR, re-amplified and transferred to vector pTRBOc as described above to produce final vector pTMV-iLOV-2A-CP_TMV_. The integrity of the vector was determined by PCR as described above.

The subgenomic expression vector was produced by amplifying the iLOV sequence using primers PacI-iLOV and iLOV-Stop-NotI. The product was transferred to vector pCR2.1-TOPO and its integrity was confirmed as described above, and then the insert was excised with PacI and NotI and transferred to vector pJL (Lindbo, 2007), which had been linearized with the same enzymes. The integrity of the resulting vector pJL-iLOV was confirmed by PCR using primers TMV5482f and TMV6269r.

In TMV-derived viral vectors, the expression of the proteins of interest is controlled by the CP subgenomic promoters. All vectors were maintained and amplified in the *E. coli* strain DH5α and their integrity was confirmed by sequencing prior to the inoculation of *N. benthamiana* plants. Full sequences of the recombinant viruses can be found online as Supplementary Material.

### Infection and Cultivation of *Nicotiana benthamiana* Plants

Three leaves from 4-week-old *N. benthamiana* plants were each inoculated with 10 μg of pTMV-CP_TMV_-iLOV, pTMV-CP_TMV_-G_4_S-iLOV, pTMV-iLOV-2A-CP_TMV_, pJL-iLOV, or pPVX-iLOV-2A-CP_PV X_. The leaf surface was gently abraded with Celite 545. After incubation for 15–20 min, the leaves were rinsed with water to remove Celite and excess DNA. The plants were cultivated in a phytochamber (12-h photoperiod, 5000–10,000 lux, 26°C/20°C light/dark temperature, humidity 70%).

### Protein Isolation and Analysis

SDS-PAGE was carried out using 12% resolving gels and a 4% stacking gels loaded with 20 μl of sample per lane. Samples for SDS-PAGE followed by western blot were prepared by extracting total protein from systemically infected leaves 1:2 in PBS, followed by centrifugation (20,000 × *g*, 15 min, 4°C) and boiling for 5 min in 5× reducing loading buffer (Laemmli, 1970). For in-gel fluorescence detection by UV light, the samples were prepared as above but were not boiled. The separated proteins were either stained with Coomassie Brilliant Blue G250 or transferred onto a Hybond C nitrocellulose membrane (GE Healthcare, Munich, Germany) using the semi-dry blotting system (BioRad, Munich, Germany). The membrane was blocked for at least 30 min in 4% skimmed milk in PBS. The iLOV protein was detected at room temperature overnight using an iLOV-specific polyclonal antibody (kindly provided by John Christie, Institute of Molecular Cell and Systems Biology, University of Glasgow, Scotland) diluted 1:4000 in PBS, followed by incubation for at least 2 h with a secondary alkaline phosphatase-conjugated goat-anti-rabbit (GAR^AP^) antibody (Dianova, Hamburg, Germany) diluted 1:5000 in PBS. The TMV and PVX were detected using a TMV-specific polyclonal antibody (Bioreba, Reinach, Switzerland) or a PVX-specific polyclonal antibody (DSMZ, Braunschweig, Germany) each diluted 1:5000 in PBS, followed by the secondary GAR^AP^ antibody as described above. The signals were visualized with nitroblue tetrazolium chloride/5-bromo-4-chloro-3-indolyphosphate (NBT/BCIP) *p*-toluidine salt (Roth, Karlsruhe, Germany). The P7712 Pre-stained Protein Standard (New England Biolabs, Ipswich, Massachusetts, USA) was used for sizing. We estimated the ratio of fusion protein and free iLOV by densitometric analysis of the αiLOV western blot by using ImageJ v1.50f software. The value of the iLOV-2A-CP_TMV_ band was divided by the density values of all bands for the fusion protein and free iLOV, multiplied by 100.

### Analysis of Viral RNA

For the sequences analysis of progeny viruses, total RNA from systemically infected *N. benthamiana* leaves was extracted using the RNeasy Plant Mini Kit (Qiagen, Hilden, Germany) according to the manufacturer’s instructions. 2 μg RNA samples were treated with 3 U of DNaseI (Thermo Fisher Scientific, Waltham, United States) prior to cDNA synthesis by M-MLV Reverse Transcriptase RNaseH Minus Point Mutant (Promega, Madison, WI, United States). 1 μg DNase-digested RNA was mixed with 0.5 μl TMV6269r primer, incubated for 10 min at 80°C and for 10 min at 4°C allowing primer annealing. For cDNA synthesis 5 μl 5x M-MLV reaction buffer, 1 mM dNTPs, 2.5 μl DEPC-H_2_O, and 1 μl M-MLV reverse were added and reverse transcription was carried out (30 min at 40°C, 20 min at 45°C, 20 min at 50°C, 20 min at 55°C, 20 min at 70°C). The integrity of CP_TMV_-iLOV, CP_TMV_-G_4_S-iLOV, iLOV-2A-CP, and iLOV was confirmed by PCR using the TMV5482f and TMV6269r primers amplifying a region between the movement protein and the CP_TMV_ coding sequence. The products were resolved by 1.2% agarose gel electrophoresis in 1x TAE buffer. Ethidium bromide was used to visualize the DNA under UV light.

### Preparation of Grids and Transmission Electron Microscopy

Pioloform-coated nickel grids (Plano, Wetzlar, Germany) were used for all preparations. For immunogold decoration the grids were incubated on a drop of systemically infected plant leaf extract for 30 min at room temperature, washed once with 20 drops of PBS containing 0.1% Tween-20 (PBST), and blocked with 0.5% bovine serum albumin (Sigma, Taufkirchen, Germany) in PBS for 20 min. After blocking, adsorbed hybrid particles were incubated for at least 2 h with the iLOV-specific antiserum described above (diluted 1:100), washed again and the particles were detected with a goat-anti-rabbit antibody conjugated to 15-nm gold particles (British BioCell, Cardiff, United Kingdom) for at least 2 h. Before staining with five drops of 1% uranyl acetate, the grids were extensively washed with PBST and twice with water. Transmission electron microscopy was carried out using a Zeiss EM10 microscope (Carl Zeiss AG, Jena, Germany).

Hybrid particles were captured from systemically infected leaf extracts by an αiLOV antibody. Therefore, the grids were incubated for 30 min on the iLOV-specific antibody, before washing and blocking with 0.5% BSA as described above. After another washing step the antibody was used to capture the TMV-iLOV particles from leaf extracts. The grids were washed and stained as described above.

## Results

### Vector Construction and Inoculation of Plants

**FIGURE 1:**
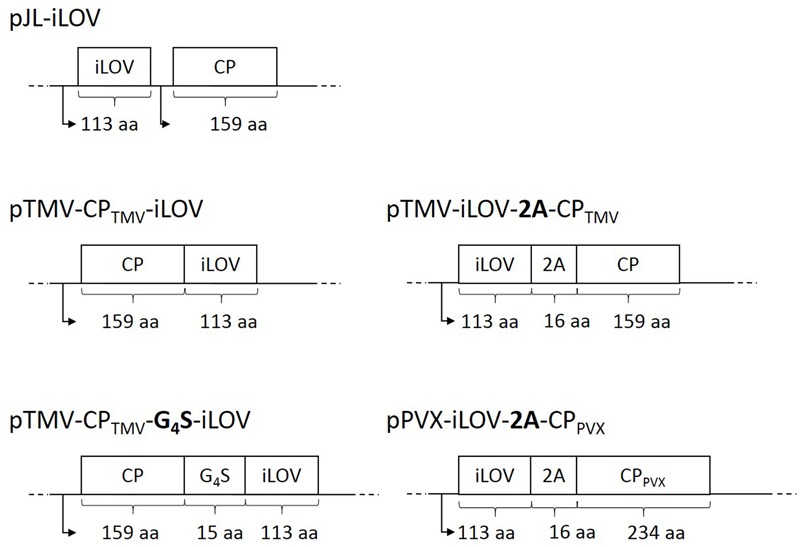
Schematic overview of TMV- and PVX-derived vectors for the expression of iLOV. The target genes are depicted as part of the full viral genomes. The iLOV sequence was added either as a direct fusion or with an intervening (G_4_S)_3_ linker to the 3′ end of the TMV CP gene. The iLOV gene was also fused via the FMDV 2A sequence to the 5′ end of the TMV CP and was expressed as a free polypeptide under the control of a subgenomic promoter (arrows). The pPVX-iLOV-2A-CP_PV X_ vector was generated by fusing the iLOV sequence via the FMDV 2A sequence to the 5′ end of the PVX CP gene. Abbreviation: aa = length of amino acid sequence.

We constructed three TMV-based vectors (**Figure 1**) in which the iLOV coding sequence was joined to the 3′ end of the TMV *cp* gene (a) directly or (b) via a glycine-serine linker. Furthermore, the sequence was fused with (c) a FMDV 2A sequence to the 5′ end of the *cp* gene to enable a ribosomal skip during translation. A fourth TMV-based vector with a CP subgenomic promoter was used to express the free iLOV protein. As a positive control the iLOV sequence was also fused to the 5′ end of the PVX CP via the FMDV 2A sequence.

**FIGURE 2:**
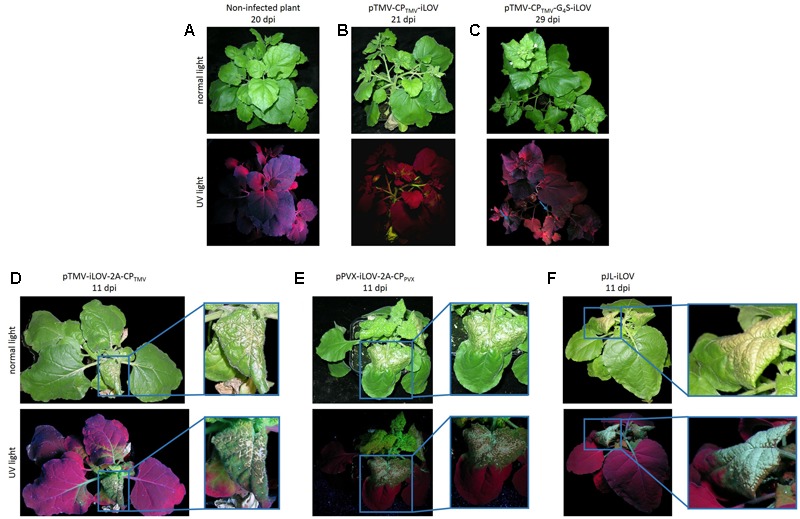
Symptoms of infection and detection of fluorescence produced in *N. benthamiana* plants inoculated with TMV- and PVX-derived vectors for the expression of iLOV. **(A)** Non-infected *N. benthamiana* plant and plants infected with **(B)** pTMV-CP_TMV_-iLOV, **(C)** pTMV-CP_TMV_-G_4_S-iLOV, **(D)** pTMV-iLOV-2A-CP_TMV_, **(E)** pPVX-iLOV-2A-CP_PV X_, and **(F)** pJL-iLOV were viewed under UV light or normal light 11–29 dpi depending on the infection progress. All plants showed evidence of systemic infection as revealed by the green fluorescence. Plants infected with pPVX-iLOV-2A-CP_PV X_, pTMV-iLOV-2A-CP_TMV_, or pJL-iLOV showed the most severe symptoms, including necrotic leaves.

The vectors were used to inoculate *Nicotiana benthamiana* plants, resulting in systemic infections in all cases as revealed by the green fluorescence of the iLOV polypeptide, even in plants inoculated with pTMV-CP_TMV_-iLOV and pTMV-CP_TMV_-G_4_S-iLOV (**Figure 2**). However, long-distant movement was delayed in plants infected with pTMV-CP_TMV_-iLOV and pTMV-CP_TMV_-G_4_S-iLOV, and the fluorescence signal was therefore present mostly in the stem whereas only small portions of leaf tissue showed iLOV fluorescence 21–29 dpi (**Figures 2B,C**). Plants infected with pTMV-iLOV-2A-CP_TMV_ and pPVX-iLOV-2A-CP_PV X_ showed systemic fluorescent spots from 6 to 7 dpi onward. In addition to the bright green fluorescence, the plants inoculated with pTMV-iLOV-2A-CP_TMV_ also showed severe symptoms of TMV infection, including mosaic-like mottling, dwarfing and necrosis at 11 dpi (**Figure 2D**). The necrotic lesions were more severe when the free iLOV polypeptide was expressed from the subgenomic promoter (**Figure 2F**).

### Analysis of Recombinant TMV Particles Displaying iLOV

Leaves were collected from plants systemically infected with pTMV-CP_TMV_-iLOV, pTMV-CP_TMV_-G_4_S-iLOV, pTMV-iLOV-2A-CP_TMV_, pJL-iLOV, and pPVX-iLOV-2A-CP_PV X_, and the modified CPs were characterized by SDS-PAGE and western blotting using antibodies specific for TMV or iLOV.

**FIGURE 3:**
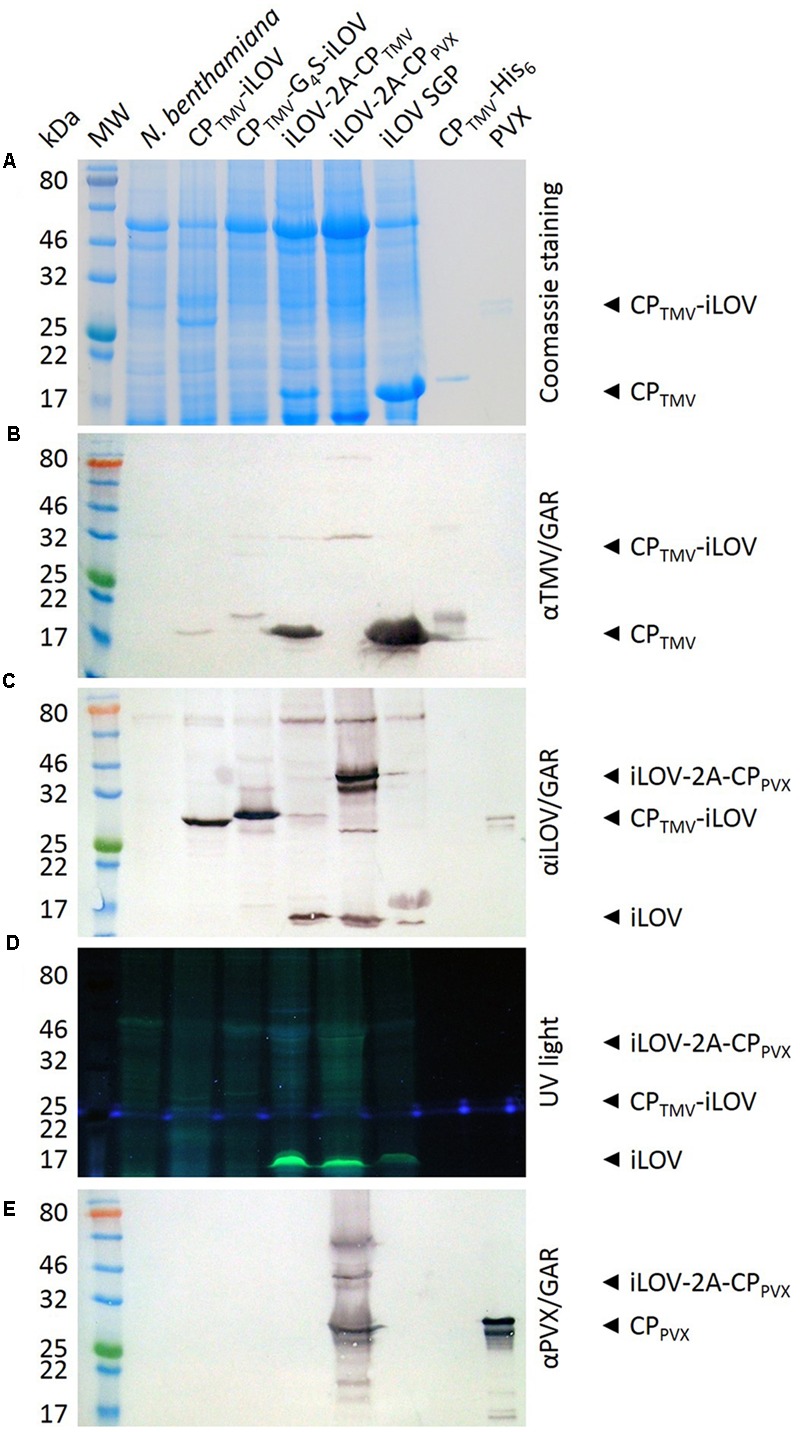
Analysis of infected *N. benthamiana* leaves expressing iLOV fusion proteins. Extracts of systemically infected leaf extracts were separated by SDS-PAGE (20-μl samples) and proteins were **(A)** stained with Coomassie Brilliant Blue G250, **(B,C,E)** transferred to a nitrocellulose membrane for western blotting, or **(D)** detected under UV light. MW = P7712 Pre-stained Protein Standard, *N. benthamiana* = extract from non-infected leaf. CP_TMV_-His = TMV CP-His (control), PVX = PVX particles. Other lanes are extracts from leaves infected with the corresponding viral vectors. Antibody detection: **(B)** TMV-specific antibody and GAR^AP^, **(C)** iLOV-specific antibody and GAR^AP^, **(E)** PVX-specific antibody and GAR^AP^.

Western blots of total leaf extracts from plants infected with pTMV-CP_TMV_-iLOV and pTMV-CP_TMV_-G_4_S-iLOV showed only faint bands at the anticipated molecular weights of 30.5 and 31.4 kDa when probed with the TMV-specific antibody (**Figure 3B**) but strong signals when probed with the iLOV-specific antibody (**Figure 3C**), probably due to epitope access and weak systemic leaf infection. The TMV-specific antibody also revealed a 17.6 kDa band corresponding to the free TMV CP (**Figure 3B**) but as expected we were unable to detect the free iLOV polypeptide, either by western blot or under UV light. Weak bands corresponding to the CP_TMV_-iLOV and CP_TMV_-G_4_S-iLOV fusion proteins were also revealed under UV light. The weakness of the signal was probably due to the high background caused by the autofluorescence of the leaf extracts (**Figure 3D**).

Western blots of total leaf extracts from plants infected with pTMV-iLOV-2A-CP_TMV_ showed only a faint band at the anticipated molecular weight of 32.5 kDa when probed with the TMV-specific antibody (**Figure 3B**) or with the iLOV-specific antibody (**Figure 3C**). As above, the TMV-specific antibody also detected a 17.6 kDa band corresponding to the free TMV CP. However, in this case the iLOV-specific antibody detected not only the fusion protein but also the 13 kDa free iLOV polypeptide (**Figure 3B**). Only the latter could be detected under UV light (**Figure 3D**). Densitometric analyses show a ratio of 2:3 for iLOV-2A-CP_TMV_ fusion protein to free iLOV.

The TMV vector, plants infected with pPVX-iLOV-2A-CP_PV X_ displayed a strong signal for the fusion protein (39.9 kDa) in western blots probed with either the PVX-specific or iLOV-specific antibodies (**Figures 3E,C**). A 25 kDa band also detected by western blot with the PVX-specific antibody represented the free PVX CP (**Figure 3E**). The 13 kDa free iLOV polypeptide was detected by western blot using the iLOV-specific antibody (**Figure 3C**) and also produced a strong signal under UV light (**Figure 3D**).

Leaf extracts from plants infected with the TMV subgenomic promoter control construct yielded strong signals for both the free TMV CP and the free iLOV polypeptide when detected with the corresponding antibodies (**Figures 3B,C**), and the iLOV polypeptide was also detected under UV light as expected (**Figure 3D**).

**FIGURE 4:**
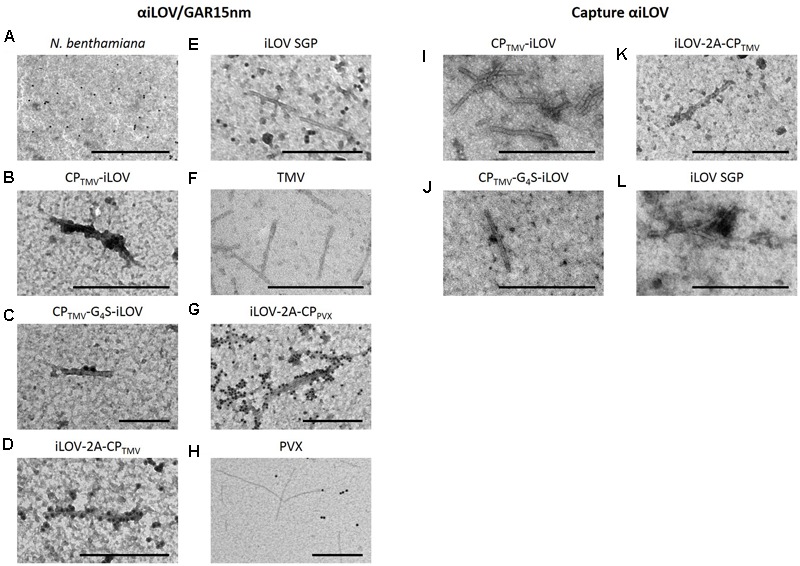
Electron micrographs of infected leaf extracts expressing recombinant TMV and PVX particles displaying iLOV. **(A–H)** Immunogold decoration: **(A)** Non-infected *N. benthamiana* leaf extract, **(B)** CP_TMV_-iLOV, **(C)** CP_TMV_-G_4_S-iLOV, **(D)** iLOV-2A-CP_TMV_, **(E)** iLOV SGP, **(F)** TMV particles, **(G)** iLOV-2A-CP_PV X_, and **(H)** PVX particles. The particles were detected with an iLOV-specific antibody and a goat anti-rabbit secondary antibody labeled with 15-nm gold. **(I–L)** Enrichment of particles by capturing with iLOV-specific antibody: **(I)** CP_TMV_-iLOV, **(J)** CP_TMV_-G_4_S-iLOV, **(K)** iLOV-2A-CP_TMV_, **(L)** iLOV SGP. Bar = **(A)** 1 μm, **(B,D–L)** = 500 nm, **(C)** = 200 nm.

Transmission electron microscopy confirmed the presence of iLOV on the surface of CP_TMV_-iLOV (**Figure 4B**), CP_TMV_-G_4_S-iLOV (**Figure 4C**), iLOV-2A-CP_TMV_ (**Figure 4D**), and iLOV-2A-CP_PV X_ particles (**Figure 4G**) due to the recognition of the iLOV polypeptide by specific antibodies conjugated to 15-nm gold particles. In contrast to the iLOV-2A-CP_TMV_ and iLOV-2A-CP_PV X_ particles, only a few decorated TMV particles were observed in plants expressing CP_TMV_-iLOV and CP_TMV_-G_4_S-iLOV. Moreover, the TMV-iLOV particles were captured from leaf extracts by the iLOV-specific antibody (**Figures 4I–K**). Although particles were not decorated in plants infected with pJL-iLOV expressing the free iLOV polypeptide from a subgenomic promoter (**Figure 4E**), some particles were captured by the iLOV antibody (**Figure 4L**), and were also detected in the western blot probed with this antibody (**Figure 3C**). TMV and PVX particles without fusion proteins were not decorated by the antibodies as expected (**Figures 4F,H**).

**FIGURE 5:**
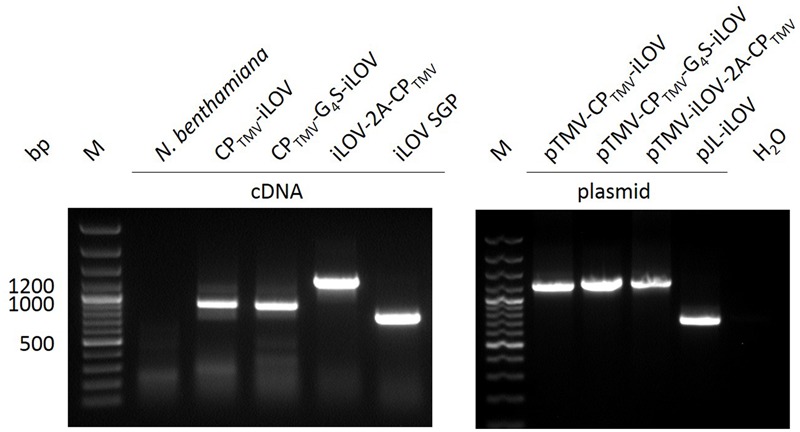
RT-PCR analysis of infected plant material expressing iLOV. RT-PCR products of isolated RNA from *N. benthamiana* (non-infected plant), and plants infected with pTMV-CP_TMV_-iLOV, pTMV-CP_TMV_-G_4_S-iLOV, pTMV-iLOV-2A-CP_TMV_, and pJL-iLOV. Plasmid DNA of the vectors serves as control in PCR. M: GeneRuler 100 bp Plus DNA Ladder.

Total RNA was isolated from *N. benthamiana* leaves systemically infected with pTMV-CP_TMV_-iLOV, pTMV-CP_TMV_-G_4_S-iLOV, pTMV-iLOV-2A-CP_TMV_, or pJL-iLOV. Non-infected leaves served as negative controls. Residual RNA was removed by RNase prior to reverse transcription of the viral RNA with a TMV specific primer. The cDNA and control plasmids were amplified by primers flanking a region between the *movement protein* and *cp* genes (**Figure 5**). pTMV-CP_TMV_-iLOV and pTMV-CP_TMV_-G_4_S-iLOV infected leaves should have yield fragments of 1216 and 1261-bp in size, but revealed only deleted versions with a size of ∼900 bp. Infections with pTMV-iLOV-2A-CP_TMV_ and pJL-iLOV indicated products of 1276 or 733 bp in RT-PCR analysis, as expected.

## Discussion

The capsids of plant viruses are widely used as building blocks for nanotechnological applications because they are suitable as scaffolds for the display of diverse functional molecules. The C-terminus of each PVX CP subunit is located within the PVX particle whereas the N-terminus projects from the outer surface. In contrast, both the N-terminus and C-terminus of each TMV CP subunit are exposed on the external surface and hence provide excellent insertion sites for the display of fusion peptides.

In this study, TMV-derived vectors were generated to display the monomeric fluorescent protein iLOV (**Figure 1**). The iLOV sequence was genetically fused to the 3′ end of the TMV CP either directly or via a glycine-serine linker, or with the FMDV 2A sequence at the 5′ end of the CP. As positive controls, iLOV was expressed as a PVX CP fusion using the FMDV 2A sequence, and as a free iLOV polypeptide under the control of a duplicated subgenomic promoter.

The iLOV polypeptide has a molecular weight of 13 kDa and is therefore even smaller than the 15.5 kDa ProtA fragment, which is known to form hybrid TMV particles efficiently when expressed as a CP fusion (Werner et al., 2006). We therefore anticipated that iLOV could be displayed on the surface of TMV, but the multiplication of the pTMV-CP_TMV_-iLOV and pTMV-CP_TMV_-G_4_S-iLOV viral vectors was inefficient, potentially due to steric hindrance that may affect particle assembly, movement or more likely phloem unloading. Although each of the vectors we tested was able to infect *N. benthamiana* plants systemically, as revealed by the green iLOV fluorescence, long-distance movement was delayed in plants inoculated with pTMV-CP_TMV_-iLOV or pTMV-CP_TMV_-G_4_S-iLOV. More precisely, in plants infected with the C-terminal iLOV direct fusion constructs, the fluorescence remained mainly in the stem, which again suggests inefficient particle assembly due to steric hindrance. The presence of peptides fused to the CP increases the genetic load and can interfere with TMV particle assembly, local and systemic movement, and in some cases can even induce the expression of host defense genes (Beachy et al., 1996; Bendahmane et al., 1999). Therefore, the characteristics of the target peptide should be tailored to facilitate particle assembly, which can be influenced by steric effects (Dawson et al., 1989) and the pI of the fusion peptide (Bendahmane et al., 1999). Peptide fusions with pI values equal to or lower than that of the wild-type TMV CP can move systemically, although additional positively charged amino acids can inhibit virus movement. This results in the selection of point mutations and deletions that restore normal virus functions, as well as the appearance of necrotic lesions in the leaves (Beachy et al., 1996; Bendahmane et al., 1999). The iLOV polypeptide comprises only ∼13% positively charged amino acids and the CP_TMV_-iLOV fusion protein has a pI of 4.8, which is comparable to the pI of wild-type CP_TMV_ (pI 4.5). The restricted systemic movement of CP_TMV_-iLOV and CP_TMV_-G_4_S-iLOV TMV particles must therefore reflect the size of the fused polypeptide.

Infected plants with severe TMV or PVX symptoms such as necrosis were observed following inoculation with pTMV-iLOV-2A-CP_TMV_, pPVX-iLOV-2A-CP_PV X_ and pJL-iLOV (**Figure 2**). This may reflect the ability of iLOV to produce ROS that can interfere with host metabolism and induce a hypersensitive response (Shu et al., 2011; Ruiz-Gonzalez et al., 2013). The iLOV polypeptide was expressed as a CP fusion protein but also as a free protein due to the use of the 2A peptide linker, allowing hybrid particles to assemble and move systemically (Cruz et al., 1996). The concentration of ROS might therefore be much higher in plants infected with pTMV-iLOV-2A-CP_TMV_, pPVX-iLOV-2A-CP_PV X_, or pJL-iLOV than those infected with pTMV-CP_TMV_-iLOV or pTMV-CP_TMV_-G_4_S-iLOV, resulting in severe symptoms such as necrotic lesions (**Figure 2**).

The expression of the CP_TMV_-iLOV and iLOV-2A-CP_TMV_ fusion proteins as well as free CP_TMV_ and free iLOV was confirmed by in-gel UV fluorescence analysis and western blots with antibodies specific for either TMV or iLOV (**Figure 3**). These data confirm that compositional limitations for fusions to the TMV CP fusion proteins can be overcome not only by inserting a read-through sequence motif at the 3′ end of the TMV *cp* gene (Turpen et al., 1995), but also by including the FMDV 2A sequence as an N-terminal fusion. pPVX-iLOV-2A-CP_PV X_ or pTMV-iLOV-2A-CP_TMV_ constructs expressed larger amounts of the free iLOV polypeptide than CP fusion proteins (**Figure 3**).

Ribosomal skip sequences are widely used with PVX vectors to facilitate the assembly of hybrid particles, as reported for fusions containing the 45 kDa VP6 rotavirus capsid protein (O’Brien et al., 2000), GFP (Cruz et al., 1996), various epitopes (Marconi et al., 2006; Zelada et al., 2006; Uhde-Holzem et al., 2010), a single-chain antibody fragment (Smolenska et al., 1998) and lipase (Carette et al., 2007). The major disadvantage of the utilization of the FMDV 2A ribosomal skip sequence is the unpredictable ratio of fusion protein to CP (Luke et al., 2010; Minskaia et al., 2013), but this can be adjusted through the use of length-optimized variants of the 2A sequence (Minskaia et al., 2013). When displaying epitopes on the surface of a virus for immunization, it is better to choose a 2A sequence variant that achieves the optimal epitope-to-CP ratio thus maximizing the number of epitopes presented to the immune system. When displaying fluorescent proteins on the surface of a virus for bioimaging applications or to track virus movement, the brightest fluorescence is achieved by maximizing the density of the fluorescent fusion, as shown for the CP_TMV_-iLOV direct fusion protein but not for the iLOV-2A-CP_TMV_ variant, which also produced ∼60% free iLOV polypeptide (**Figure 3**).

Transmission electron microscopy (**Figure 4**) confirmed the assembly of TMV particles displaying the iLOV polypeptide. Hybrid particles displaying iLOV were similar in shape and size to wild-type TMV particles, which are filamentous, rigid rods 300 nm in length. The hybrid particles were recognized by the iLOV-specific antibody which was detected in turn using a secondary antibody conjugated to 15-nm gold particles. Only a few particles were detected in leaves from plants expressing the CP_TMV_-iLOV and CP_TMV_-G_4_S-iLOV constructs, probably because most of the fluorescence (and therefore most of the hybrid virus particles) was retained in the stems. Another likely explanation could be the loss of the inserted iLOV sequence in the direct *cp* gene fusions, as indicated by RNA analysis and the detection of CP (**Figures 2B, 5**). Although the CP_TMV_-iLOV and CP_TMV_-G_4_S-iLOV viruses seem to be able to replicate, they could not move efficiently from cell-to-cell. There might be also mostly unmodified CP for the assembly available, but it seems not to be enough to compensate the sterically hindrance arising from the CP-iLOV fusion proteins. It can thus be speculated, that the remaining modification is detrimental to particle assembly, movement or phloem unloading. This might also be a possible explanation for the little CP_TMV_-iLOV and CP_TMV_-G_4_S-iLOV particles observed in electron micrographs. Gold labeling was especially prevalent for the iLOV-2A-CP_TMV_ and iLOV-2A-CP_PV X_ particles, indicating the incorporation of iLOV-2A-CP fusion proteins during virion assembly. Our findings therefore confirm that TMV vectors containing the FMDV 2A sequence are suitable for the production of hybrid TMV particles displaying proteins with a molecular weight of at least 13 kDa, and that the corresponding free polypeptide is also expressed at high levels. This strategy also means that not only the C-terminus, but also the N-terminus of the TMV CP becomes suitable for peptide display providing a nano-addressable virus scaffold.

## Permission to Reuse and Copyright

Permission must be obtained for use of copyrighted material from other sources (including the web). Please note that it is compulsory to follow figure instructions.

## Author Contributions

UC and JR provided the idea of the work and designed the experiments. JR conducted the experiments. UC, JR, and RF participated in the interpretation of results and critically reviewed the manuscript. JR wrote the paper. All authors read and approved the final manuscript.

## Conflict of Interest Statement

The authors declare that the research was conducted in the absence of any commercial or financial relationships that could be construed as a potential conflict of interest.

## Abbreviations

CP: coat protein
CPMV: Cowpea mosaic virus
dpi: days post-inoculation
FMDV: Foot-and-mouth disease virus
G_4_S: peptide linker consisting of four glycines and one serine, repeated 3 times
GAR^AP^: secondary alkaline phosphatase-conjugated goat-anti-rabbit antibody
GFP: green fluorescent protein
pI: isoelectric point
PBS: phosphate buffered saline
PVX: Potato virus X
ROS: reactive oxygen species
SGP: subgenomic promoter
TMV: Tobacco mosaic virus

## References

[B1] Azucena C. Eber F. J. Trouillet V. Hirtz M. Heissler S. Franzreb M. (2012). New approaches for bottom-up assembly of tobacco mosaic virus-derived nucleoprotein tubes on defined patterns on silica- and polymer-based substrates. *Langmuir* 28 14867–14877. 10.1021/la302774h 22950722

[B2] Beachy R. N. Fitchen J. H. Hein M. B. (1996). Use of plant viruses for delivery of vaccine epitopes. *Ann. N. Y. Acad. Sci.* 792 43–49. 10.1111/j.1749-6632.1996.tb32489.x 8678419 PMC7167674

[B3] Bendahmane M. Koo M. Karrer E. Beachy R. N. (1999). Display of epitopes on the surface of tobacco mosaic virus: impact of charge and isoelectric point of the epitope on virus-host interactions. *J. Mol. Biol.* 290 9–20. 10.1006/jmbi.1999.2860 10388554 PMC7126444

[B4] Bloomer A. C. Champness J. N. Bricogne G. Staden R. Klug A. (1978). Protein disk of tobacco mosaic virus at 2.8 A resolution showing the interactions within and between subunits. *Nature* 276 362–368. 10.1038/276362a0 19711551

[B5] Blum A. S. Soto C. M. Wilson C. D. Brower T. L. Pollack S. K. Schull T. L. (2005). An engineered virus as a scaffold for three-dimensional self-assembly on the nanoscale. *Small* 1 702–706. 10.1002/smll.200500021 17193509

[B6] Brown A. D. Naves L. Wang X. Ghodssi R. Culver J. N. (2013). Carboxylate-directed in vivo assembly of virus-like nanorods and tubes for the display of functional peptides and residues. *Biomacromolecules* 14 3123–3129. 10.1021/bm400747k 23883304

[B7] Bruckman M. A. Kaur G. Lee L. A. Xie F. Sepulveda J. Breitenkamp R. (2008). Surface modification of tobacco mosaic virus with ”click” chemistry. *Chembiochem* 9 519–523. 10.1002/cbic.200700559 18213566

[B8] Bruckman M. A. Steinmetz N. F. (2014). Chemical modification of the inner and outer surfaces of Tobacco Mosaic Virus (TMV). *Methods Mol. Biol.* 1108 173–185. 10.1007/978-1-62703-751-8_13 24243249 PMC4546836

[B9] Brunel F. M. Lewis J. D. Destito G. Steinmetz N. F. Manchester M. Stuhlmann H. (2010). Hydrazone ligation strategy to assemble multifunctional viral nanoparticles for cell imaging and tumor targeting. *Nano Lett.* 10 1093–1097. 10.1021/nl1002526 20163184 PMC3988696

[B10] Carette N. Engelkamp H. Akpa E. Pierre S. J. Cameron N. R. Christianen P. C. (2007). A virus-based biocatalyst. *Nat. Nanotechnol.* 2 226–229. 10.1038/nnano.2007.76 18654267

[B11] Chapman S. Faulkner C. Kaiserli E. Garcia-Mata C. Savenkov E. I. Roberts A. G. (2008). The photoreversible fluorescent protein iLOV outperforms GFP as a reporter of plant virus infection. *Proc. Natl. Acad. Sci. U.S.A.* 105 20038–20043. 10.1073/pnas.0807551105 19060199 PMC2604982

[B12] Chatterji A. Ochoa W. F. Ueno T. Lin T. Johnson J. E. (2005). A virus-based nanoblock with tunable electrostatic properties. *Nano Lett.* 5 597–602. 10.1021/nl048007s 15826093

[B13] Christie J. M. Hitomi K. Arvai A. S. Hartfield K. A. Mettlen M. Pratt A. J. (2012). Structural tuning of the fluorescent protein iLOV for improved photostability. *J. Biol. Chem.* 287 22295–22304. 10.1074/jbc.M111.318881 22573334 PMC3381190

[B14] Cruz S. S. Chapman S. Roberts A. G. Roberts I. M. Prior D. A. Oparka K. J. (1996). Assembly and movement of a plant virus carrying a green fluorescent protein overcoat. *Proc. Natl. Acad. Sci. U.S.A.* 93 6286–6290. 10.1073/pnas.93.13.6286 8692807 PMC39014

[B15] Dawson W. O. Lewandowski D. J. Hilf M. E. Bubrick P. Raffo A. J. Shaw J. J. (1989). A tobacco mosaic virus-hybrid expresses and loses an added gene. *Virology* 172 285–292. 10.1016/0042-6822(89)90130-X 2773319

[B16] Donnelly M. L. Hughes L. E. Luke G. Mendoza H. Ten Dam E. Gani D. (2001a). The ’cleavage’ activities of foot-and-mouth disease virus 2A site-directed mutants and naturally occurring ’2A-like’ sequences. *J. Gen. Virol.* 82 1027–1041. 11297677 10.1099/0022-1317-82-5-1027

[B17] Donnelly M. L. Luke G. Mehrotra A. Li X. Hughes L. E. Gani D. (2001b). Analysis of the aphthovirus 2A/2B polyprotein ’cleavage’ mechanism indicates not a proteolytic reaction, but a novel translational effect: a putative ribosomal ’skip’. *J. Gen. Virol.* 82 1013–1025. 11297676 10.1099/0022-1317-82-5-1013

[B18] Dujardin E. Peet C. Stubbs G. Culver J. N. Mann S. (2003). Organization of metallic nanoparticles using tobacco mosaic virus templates. *Nano Lett.* 3 413–417. 10.1021/nl034004o

[B19] Gawthorne J. A. Reddick L. E. Akpunarlieva S. N. Beckham K. S. Christie J. M. Alto N. M. (2012). Express your LOV: an engineered flavoprotein as a reporter for protein expression and purification. *PLoS ONE* 7:e52962 10.1371/journal.pone.0052962 PMC3531456 23300834

[B20] Klug A. (1999). The tobacco mosaic virus particle: structure and assembly. *Philos. Trans. R. Soc. Lond. B Biol. Sci.* 354 531–535. 10.1098/rstb.1999.0404 10212932 PMC1692534

[B21] Laemmli U. K. (1970). Cleavage of structural proteins during the assembly of the head of bacteriophage T4. *Nature* 227 680–685. 10.1038/227680a0 5432063

[B22] Lee K. L. Hubbard L. C. Hern S. Yildiz I. Gratzl M. Steinmetz N. F. (2013). Shape matters: the diffusion rates of TMV rods and CPMV icosahedrons in a spheroid model of extracellular matrix are distinct. *Biomater. Sci.* 1 581–588. 10.1039/C3BM00191A PMC3824242 24244867

[B23] Lee K. L. Uhde-Holzem K. Fischer R. Commandeur U. Steinmetz N. F. (2014). Genetic engineering and chemical conjugation of potato virus X. *Methods Mol. Biol.* 1108 3–21. 10.1007/978-1-62703-751-8_1 24243237 PMC5207041

[B24] Lewis J. D. Destito G. Zijlstra A. Gonzalez M. J. Quigley J. P. Manchester M. (2006). Viral nanoparticles as tools for intravital vascular imaging. *Nat. Med.* 12 354–360. 10.1038/nm1368 16501571 PMC2536493

[B25] Lindbo J. A. (2007). TRBO: a high-efficiency tobacco mosaic virus RNA-based overexpression vector. *Plant Physiol.* 145 1232–1240. 10.1104/pp.107.106377 17720752 PMC2151719

[B26] Liu N. Wang C. Zhang W. Luo Z. Tian D. Zhai N. (2012). Au nanocrystals grown on a better-defined one-dimensional tobacco mosaic virus coated protein template genetically modified by a hexahistidine tag. *Nanotechnology* 23 335602 10.1088/0957-4484/23/33/335602 22842556

[B27] Luckanagul J. Lee L. A. Nguyen Q. L. Sitasuwan P. Yang X. Shazly T. (2012). Porous alginate hydrogel functionalized with virus as three-dimensional scaffolds for bone differentiation. *Biomacromolecules* 13 3949–3958. 10.1021/bm301180c 23148483

[B28] Luke G. Escuin H. De Felipe P. Ryan M. (2010). 2A to the fore - research, technology and applications. *Biotechnol. Genet. Eng. Rev.* 26 223–260. 10.5661/bger-26-223 21415883

[B29] Manchester M. Singh P. (2006). Virus-based nanoparticles (VNPs): platform technologies for diagnostic imaging. *Adv. Drug Deliv. Rev.* 58 1505–1522. 10.1016/j.addr.2006.09.014 17118484

[B30] Marconi G. Albertini E. Barone P. De Marchis F. Lico C. Marusic C. (2006). In planta production of two peptides of the Classical Swine Fever Virus (CSFV) E2 glycoprotein fused to the coat protein of potato virus X. *BMC Biotechnol.* 6:29 10.1186/1472-6750-6-29 PMC1534020 16792815

[B31] McCormick A. A. Corbo T. A. Wykoff-Clary S. Nguyen L. V. Smith M. L. Palmer K. E. (2006). TMV-peptide fusion vaccines induce cell-mediated immune responses and tumor protection in two murine models. *Vaccine* 24 6414–6423. 10.1016/j.vaccine.2006.06.003 16860441

[B32] Meunier S. Strable E. Finn M. G. (2004). Crosslinking of and coupling to viral capsid proteins by tyrosine oxidation. *Chem. Biol.* 11 319–326. 10.1016/j.chembiol.2004.02.019 15123261

[B33] Minskaia E. Nicholson J. Ryan M. D. (2013). Optimisation of the foot-and-mouth disease virus 2A co-expression system for biomedical applications. *BMC Biotechnol.* 13:67 10.1186/1472-6750-13-67 PMC3765190 23968294

[B34] Niu Z. Bruckman M. A. Li S. Lee L. A. Lee B. Pingali S. V. (2007). Assembly of tobacco mosaic virus into fibrous and macroscopic bundled arrays mediated by surface aniline polymerization. *Langmuir* 23 6719–6724. 10.1021/la070096b 17474763

[B35] O’Brien G. J. Bryant C. J. Voogd C. Greenberg H. B. Gardner R. C. Bellamy A. R. (2000). Rotavirus VP6 expressed by PVX vectors in *Nicotiana benthamiana* coats PVX rods and also assembles into viruslike particles. *Virology* 270 444–453. 10.1006/viro.2000.0314 10793003

[B36] Royston E. Ghosh A. Kofinas P. Harris M. T. Culver J. N. (2008). Self-assembly of virus-structured high surface area nanomaterials and their application as battery electrodes. *Langmuir* 24 906–912. 10.1021/la7016424 18154364

[B37] Ruiz-Gonzalez R. Cortajarena A. L. Mejias S. H. Agut M. Nonell S. Flors C. (2013). Singlet oxygen generation by the genetically encoded tag miniSOG. *J. Am. Chem. Soc.* 135 9564–9567. 10.1021/ja4020524 23781844

[B38] Shaner N. C. Patterson G. H. Davidson M. W. (2007). Advances in fluorescent protein technology. *J. Cell Sci.* 120 4247–4260. 10.1242/jcs.005801 18057027

[B39] Shu X. Lev-Ram V. Deerinck T. J. Qi Y. Ramko E. B. Davidson M. W. (2011). A genetically encoded tag for correlated light and electron microscopy of intact cells, tissues, and organisms. *PLoS Biol.* 9:e1001041 10.1371/journal.pbio.1001041 PMC3071375 21483721

[B40] Shukla S. Ablack A. L. Wen A. M. Lee K. L. Lewis J. D. Steinmetz N. F. (2013). Increased tumor homing and tissue penetration of the filamentous plant viral nanoparticle Potato virus X. *Mol. Pharm.* 10 33–42. 10.1021/mp300240m 22731633 PMC3482416

[B41] Shukla S. Dickmeis C. Nagarajan A. S. Fischer R. Commandeur U. Steinmetz N. F. (2014). Molecular farming of fluorescent virus-based nanoparticles for optical imaging in plants, human cells and mouse models. *Biomater. Sci.* 2 784–797. 10.1039/c3bm60277j 32481848

[B42] Skuzeski J. M. Nichols L. M. Gesteland R. F. Atkins J. F. (1991). The signal for a leaky UAG stop codon in several plant viruses includes the two downstream codons. *J. Mol. Biol.* 218 365–373. 10.1016/0022-2836(91)90718-L 2010914

[B43] Smolenska L. Roberts I. M. Learmonth D. Porter A. J. Harris W. J. Wilson T. M. (1998). Production of a functional single chain antibody attached to the surface of a plant virus. *FEBS Lett.* 441 379–382. 10.1016/S0014-5793(98)01586-5 9891975

[B44] Steinmetz N. F. Lomonossoff G. P. Evans D. J. (2006a). Cowpea mosaic virus for material fabrication: addressable carboxylate groups on a programmable nanoscaffold. *Langmuir* 22 3488–3490. 16584217 10.1021/la060078e

[B45] Steinmetz N. F. Lomonossoff G. P. Evans D. J. (2006b). Decoration of cowpea mosaic virus with multiple, redox-active, organometallic complexes. *Small* 2 530–533. 10.1002/smll.200500453 17193081

[B46] Turpen T. H. Reinl S. J. Charoenvit Y. Hoffman S. L. Fallarme V. Grill L. K. (1995). Malarial epitopes expressed on the surface of recombinant tobacco mosaic virus. *Biotechnology (N Y)* 13 53–57. 9634749 10.1038/nbt0195-53

[B47] Uhde-Holzem K. Mcburney M. Tiu B. D. Advincula R. C. Fischer R. Commandeur U. (2015). Production of Immunoabsorbent nanoparticles by displaying single-domain protein A on Potato Virus X. *Macromol. Biosci.* 16 231–241. 10.1002/mabi.201500280 26440117

[B48] Uhde-Holzem K. Schlosser V. Viazov S. Fischer R. Commandeur U. (2010). Immunogenic properties of chimeric potato virus X particles displaying the hepatitis C virus hypervariable region I peptide R9. *J. Virol. Methods* 166 12–20. 10.1016/j.jviromet.2010.01.017 20138085

[B49] van Belkum A. Abrahams J. P. Pleij C. W. Bosch L. (1985). Five pseudoknots are present at the 204 nucleotides long 3′ noncoding region of tobacco mosaic virus RNA. *Nucleic Acids Res.* 13 7673–7686. 10.1093/nar/13.21.7673 3934645 PMC322079

[B50] Wen A. M. Shukla S. Saxena P. Aljabali A. A. Yildiz I. Dey S. (2012). Interior engineering of a viral nanoparticle and its tumor homing properties. *Biomacromolecules* 13 3990–4001. 10.1021/bm301278f 23121655 PMC3525095

[B51] Werner S. Marillonnet S. Hause G. Klimyuk V. Gleba Y. (2006). Immunoabsorbent nanoparticles based on a tobamovirus displaying protein A. *Proc. Natl. Acad. Sci. U.S.A.* 103 17678–17683. 10.1073/pnas.0608869103 17090664 PMC1635023

[B52] Zelada A. M. Calamante G. De La Paz Santangelo M. Bigi F. Verna F. Mentaberry A. (2006). Expression of tuberculosis antigen ESAT-6 in *Nicotiana tabacum* using a potato virus X-based vector. *Tuberculosis* 86 263–267. 10.1016/j.tube.2006.01.003 16644283

[B53] Zimmern D. (1977). The nucleotide sequence at the origin for assembly on tobacco mosaic virus RNA. *Cell* 11 463–482. 10.1016/0092-8674(77)90065-4 884732

