# Supplementary material for: Adoption of the 2A Ribosomal Skip Principle to Tobacco Mosaic Virus for Peptide Display
Source: Front Plant Sci. 2017 Jun 28;8:1125. doi: 10.3389/fpls.2017.01125 (PMC5487473; doi:10.3389/fpls.2017.01125)
Supplement: Supplementary file 1 [file Data_Sheet_1.PDF]

Supplementary Material

Adoption of the 2A ribosomal skip principle to Tobacco mosaic virus for peptide display

Juliane Röder[1], Rainer Fischer[1], Ulrich Commandeur[1*]

[1]Institute for Molecular Biotechnology, RWTH Aachen University, Aachen, Germany

*** Correspondence:**

Ulrich Commandeur: Ulrich.Commandeur@molbiotech.rwth-aachen.de

1 Nucleotide sequences of recombinant viruses

Sequences of recombinant clones (upper case) with additional upstream sequences from CP subgenomic promotors (Goelet et al., 1982;Kim and Hemenway, 1999) (lower case). Black indicates the coat protein, the glycine-serine linker is underlined, red indicates the 2A sequence, green indicates iLOV, and grey indicates a non-coding region with an additional subgenomic promoter. The vectors pTMV-CP$_{TMV}$-iLOV, pTMV-CP$_{TMV}$-G$_4$S-iLOV and pTMV-iLOV-2A-CP$_{TMV}$ are derived from pTRBOc (Lindbo, 2007). The vector pTMV-iLOV is derived from JL (Lindbo, 2007). The vector pPVX-iLOV-2A-CP$_{PVX}$ is derived from pTCXIIc (Shukla et al., 2014).

pTMV-CP$_{TMV}$-iLOV

gatcttacagtatcactactccatctcagttcgtgttcttgtcattaattaaATGCCTTATACAATC
AACTCTCCGAGCCAATTTGTTTACTTAAGTTCCGCTTATGCAGATCCTGTGCAGCTGATCAATCTGT
GTACAAATGCATTGGGTAACCAGTTTCAAACGCAACAAGCTAGGACAACAGTCCAACAGCAATTTGC
GGATGCCTGGAAACCTGTGCCTAGTATGACAGTGAGATTTCCTGCATCGGATTTCTATGTGTATAGA
TATAATTCGACGCTTGATCCGTTGATCACGGCGTTATTAAATAGCTTCGATACTAGAAATAGAATAA
TAGAGGTTGATAATCAACCCGCACCGAATACTACTGAAATCGTTAACGCGACTCAGAGGGCAGACGA
TGCGACTGTAGCTATAAGGGCTTCAATCAATAATTTGGCTAATGAACTGGTTCGTGGAACTGGCATG
TTCAATCAAGCAAGCTTTGAGACTGCTAGTGGACTTGTCTGGACCACAACTCCGGCTACTGCAAGCA
TAGAGAAGAATTTCGTCATCACTGATCCTAGGCTTCCCGATAATCCCATTATCTTTGCATCAGACGG
CTTTCTTGAATTGACAGAGTATTCGCGCGAGGAAATATTGGGAGAAATGCCCGGTTTCTTCAGGGG
CCAGAGACAGATCAAGCGACTGTCCAGAAGATAAGAGACGCAATTAGAGATCAGAGGGAGACTACTG
TGCAGTTGATAAACTACACTAAAAGCGGAAAGAAATTCTGGAACTTACTCCACCTGCAACCTGTGCG
TGATCAGAAGGGAGAGCTTCAATACTTCATCGGTGTGCAGCTCGATGGAAGTGATCATGTA

pTMV-CP$_{TMV}$-G$_4$S-iLOV

gatcttacagtatcactactccatctcagttcgtgttcttgtcattaattaaATGCCTTATACAATC
AACTCTCCGAGCCAATTTGTTTACTTAAGTTCCGCTTATGCAGATCCTGTGCAGCTGATCAATCTGT
GTACAAATGCATTGGGTAACCAGTTTCAAACGCAACAAGCTAGGACAACAGTCCAACAGCAATTTGC
GGATGCCTGGAAACCTGTGCCTAGTATGACAGTGAGATTTCCTGCATCGGATTTCTATGTGTATAGA
TATAATTCGACGCTTGATCCGTTGATCACGGCGTTATTAAATAGCTTCGATACTAGAAATAGAATAA
TAGAGGTTGATAATCAACCCGCACCGAATACTACTGAAATCGTTAACGCGACTCAGAGGGCAGACGA
TGCGACTGTAGCTATAAGGGCTTCAATCAATAATTTGGCTAATGAACTGGTTCGTGGAACTGGCATG
TTCAATCAAGCAAGCTTTGAGACTGCTAGTGGACTTGTCTGGACCACAACTCCGGCTACTGGAGGTG
GAGGTAGCGGCGGTGGAGGGAGTGGTGGAGGCGGTAGCGCAAGCATAGAGAAGAATTTCGTCATCAC
TGATCCTAGGCTTCCCGATAATCCCATTATCTTTGCATCAGACGGCTTTCTTGAATTGACAGAGTAT
TCGCGCGAGGAAATATTGGGAGAAATGCCCGGTTTCTTCAGGGGCCAGAGACAGATCAAGCGACTG
TCCAGAAGATAAGAGACGCAATTAGAGATCAGAGGGAGACTACTGTGCAGTTGATAAACTACACTAA
AAGCGGAAAGAAATTCTGGAACTTACTCCACCTGCAACCTGTGCGTGATCAGAAGGGAGAGCTTCAA
TACTTCATCGGTGTGCAGCTCGATGGAAGTGATCATGTA

pTMV-iLOV-2A-CP$_{TMV}$

gatcttacagtatcactactccatctcagttcgtgttcttgtcattaattaaATGGCAAGCATAGAG
AAGAATTTCGTCATCACTGATCCTAGGCTTCCCGATAATCCCATTATCTTTGCATCAGACGGCTTTC
TTGAATTGACAGAGTATTCGCGCGAGGAAATATTGGGGAGAAATGCCCGGTTTCTTCAGGGGCCAGA
GACAGATCAAGCGACTGTCCAGAAGATAAGAGACGCAATTAGAGATCAGAGGGAGACTACTGTGCAG
TTGATAAACTACACTAAAAGCGGAAAGAAATTCTGGAACTTACTCCACCTGCAACCTGTGCGTGATC
AGAAGGGAGAGCTTCAATACTTCATCGGTGTGCAGCTCGATGGAAGTGATCATGTATCCGGATCTAG
AAATTTTGACCTTCTTAAGCTTGCGGGAGACGTCGAGTCCAACCCCGGGCCTTATACAATCAACTCT
CCGAGCCAATTTGTTTACTTAAGTTCCGCTTATGCAGATCCTGTGCAGCTGATCAATCTGTGTACAA
ATGCATTGGGTAACCAGTTTCAAACGCAACAAGCTAGGACAACAGTCCAACAGCAATTTGCGGATGC
CTGGAAACCTGTGCCTAGTATGACAGTGAGATTTCCTGCATCGGATTTCTATGTGTATAGATATAAT
TCGACGCTTGATCCGTTGATCACGGCGTTATTAAATAGCTTCGATACTAGAAATAGAATAATAGAGG
TTGATAATCAACCCGCACCGAATACTACTGAAATCGTTAACGCGACTCAGAGGGCAGACGATGCGAC
TGTAGCTATAAGGGCTTCAATCAATAATTTGGCTAATGAACTGGTTCGTGGAACTGGCATGTTCAAT
CAAGCAAGCTTTGAGACTGCTAGTGGACTTGTCTGGACCACAACTCCGGCTACT

pTMV-iLOV

gatcttacagtatcactactccatctcagttcgtgttcttgtcattaattaaATGGCAAGCATAGAG
AAGAATTTCGTCATCACTGATCCTAGGCTTCCCGATAATCCCATTATCTTTGCATCAGACGGCTTTC
TTGAATTGACAGAGTATTCGCGCGAGGAAATATTGGGGAGAAATGCCCGGTTTCTTCAGGGGCCAGA
GACAGATCAAGCGACTGTCCAGAAGATAAGAGACGCAATTAGAGATCAGAGGGAGACTACTGTGCAG
TTGATAAACTACACTAAAAGCGGAAAGAAATTCTGGAACTTACTCCACCTGCAACCTGTGCGTGATC
AGAAGGGAGAGCTTCAATACTTCATCGGTGTGCAGCTCGATGGAAGTGATCATGTAtaagcggccgc
tcgaggggtagtcaagatgcataataaataacggattgtgtccgtaatcacacgtggtgcgtacgat
aacgcatagtgtttttccctccacttaaatcgaagggttgtgtcttggatcgcgcgggtcaaatgta
tatggttcatatacatccgcaggcacgtaataaagcgaggggttcgggtcgaggtcggctgtgaaac
tcgaaaaggttccggaaaacaaaaaagagagtggtaggtaatagtgttaataataagaaaataaata
atagtggtaagaaaggtttgaaagttgaggaaattgaggataatgtaagtgatgacgagtctatcgc
gtcatcgagtacgttttaatcaatATGCCTTATACAATCAACTCTCCGAGCCAATTTGTTTACTTAA
GTTCCGCTTATGCAGATCCTGTGCAGCTGATCAATCTGTGTACAAATGCATTGGGTAACCAGTTTCA
AACGCAACAAGCTAGGACAACAGTCCAACAGCAATTTGCGGATGCCTGGAAACCTGTGCCTAGTATG
ACAGTGAGATTTCCTGCATCGGATTTCTATGTGTATAGATATAATTCGACGCTTGATCCGTTGATCA
CGGCGTTATTAAATAGCTTCGATACTAGAAATAGAATAATAGAGGTTGATAATCAACCCGCACCGAA
TACTACTGAAATCGTTAACGCGACTCAGAGGGTAGACGATGCGACTGTAGCTATAAGGGCTTCAATC
AATAATTTGGCTAATGAACTGGTTCGTGGAACTGGCATGTTCAATCAAGCAAGCTTTGAGACTGCTA
GTGGACTTGTCTGGACCACAACTCCGGCTACT

pPVX-iLOV-2A-CP$_{PVX}$

cgaaagaggtcagcaccagctagcATGGCAAGCATAGAGAAGAATTTCGTCATCACTGATCCTAGGC
TTCCCGATAATCCCATTATCTTTGCATCAGACGGCTTTCTTGAATTGACAGAGTATTCGCGCGAGGA
AATATTGGGGAGAAATGCCCGGTTTCTTCAGGGGCCAGAGACAGATCAAGCGACTGTCCAGAAGATA
AGAGACGCAATTAGAGATCAGAGGGAGACTACTGTGCAGTTGATAAACTACACTAAAAGCGGAAAGA
AATTCTGGAACTTACTCCACCTGCAACCTGTGCGTGATCAGAAGGGAGAGCTTCAATACTTCATCGG

```
TGTGCAGCTCGATGGAAGTGATCATGTATCCGGATCTAGAAATTTTGACCTTCTTAAGCTTGCGGGA
GACGTCGAGTCCAACCCCGGGCCCGCGAGCACAACACAGCCCATAGGGTCAACTACCTCAACTACCA
CAAAAACTGCAGGCGCAACTCCTGCCACAGCTTCAGGCCTGTTCACTATCCCGGATGGGGATTTCTT
TAGTACAGCCCGTGCCATAGTAGCCAGCAATGCTGTCGCAACAAATGAGGACCTCAGCAAGATTGAG
GCTATTTGGAAGGACATGAAGGTGCCCACAGACACTATGGCACAGGCTGCTTGGGACTTAGTCAGAC
ACTGTGCTGATGTAGGATCATCCGCTCAAACAGAAATGATAGATACAGGTCCCTATTCCAACGGCAT
CAGCAGAGCTAGACTGGCAGCAGCAATTAAAGAGGTGTGCACACTTAGGCAATTTTGCATGAAGTAT
GCCCCAGTGGTATGGAACTGGATGTTAACTAACAACAGTCCACCTGCTAACTGGCAAGCACAAGGTT
TCAAGCCTGAGCACAAATTCGCTGCATTCGACTTCTTCAATGGAGTCACCAACCCAGCTGCCATCAT
GCCCAAAGAGGGGCTCATCCGGCCACCGTCTGAAGCTGAAATGAATGCTGCCCAAACTGCTGCCTTT
GTGAAGATTACAAAGGCCAGGGCACAATCCAACGACTTTGCCAGCCTAGATGCAGCTGTCACTCGAG
GTCGTATCACTGGAACAACAACCGCTGAGGCTGTTGTCACTCTACCACCACCA
```

2 References

Goelet, P., Lomonossoff, G.P., Butler, P.J., Akam, M.E., Gait, M.J., and Karn, J. (1982). Nucleotide sequence of tobacco mosaic virus RNA. *Proc Natl Acad Sci U S A* 79**,** 5818-5822.

Kim, K.H., and Hemenway, C.L. (1999). Long-distance RNA-RNA interactions and conserved sequence elements affect potato virus X plus-strand RNA accumulation. *RNA* 5**,** 636-645.

Lindbo, J.A. (2007). TRBO: a high-efficiency tobacco mosaic virus RNA-based overexpression vector. *Plant Physiol* 145**,** 1232-1240.

Shukla, S., Dickmeis, C., Nagarajan, A.S., Fischer, R., Commandeur, U., and Steinmetz, N.F. (2014). Molecular farming of fluorescent virus-based nanoparticles for optical imaging in plants, human cells and mouse models. *Biomaterials Science* 2**,** 784-797.
